# Robotic single-port femoral artery dissection and anastomosis with the da Vinci system: a cadaveric feasibility study

**DOI:** 10.1007/s11701-025-02712-8

**Published:** 2025-08-31

**Authors:** Ashley J. Williamson, Olga Greenberg, Glenn Stante, Hubert Stein, Ross Milner

**Affiliations:** 1 Section of Vascular Surgery, The University of Chicago Medicine, Chicago, IL USA; 2 Intuitive Surgical Inc., Sunnyvale, CA USA

**Keywords:** Robotic, Vascular surgery, Vascular anastomosis, Surgical site infection

## Abstract

Vascular surgical site infections (SSI) are common and associated with graft infection, surgical reintervention, and increased lengths of stay. While antibiotic prophylaxis and negative pressure dressings have improved SSI rates, reported incidence remains as high as 30%. Robotic approaches have decreased surgical site infections in multiple surgical specialties, but remain without a vascular surgery indication. We propose a novel method for robotic groin dissection and anastomosis using a single-port placement from the mid-thigh to eliminate a femoral incision. A single-port robotic platform (da Vinci SP surgical system) was used to perform femoral groin dissection and graft anastomosis from a 3 cm incision at the mid-thigh, 15 cm from the inguinal ligament in two cadaver models. Femoral dissection and anastomosis were feasible in all attempts by a novice robotic surgeon with the approach. Dissection was completed to the level of the inguinal ligament with isolation and vessel loop control of the inferior epigastric, lateral circumflex, common femoral, superficial femoral, and profunda femoris arteries. Time from dissection to creation of arteriotomy ranged from 152 to 55 min with a 97-min reduction between the first and second procedure. Creation of the vascular anastomosis with 6 mm ringed PTFE ranged from 41 to 45 min. Robotic single-port groin dissection and vascular anastomosis are feasible from a mid-thigh approach. Beginner robotic surgeons can demonstrate an efficient learning curve with quick reduction in operating time between attempts. This novel technique has the potential to reduce groin infections and improve surgical outcomes.

## Introduction and Indications

Surgical site infections in vascular surgery are associated with significant morbidity and cost. Unlike other surgical site infections, occurrence in vascular surgery leads to vascular anastomotic involvement with concern for catastrophic blowout, graft infection, and costly muscle flaps with long lengths of stay. While the incidence of this is difficult to fully appreciate, studies report infection rates as high as 10–30% [1, 2]. Consistently, lower extremity bypass surgery for limb salvage has the highest rate of SSI incidence of vascular procedures [3]. The typical exposure for a femoral anastomosis occurs at the level of the inguinal crease: an area highly colonized with bacteria. Preventative treatments to reduce surgical site infections have largely focused on pre-operative antibiotic treatment and closed incision negative pressure dressings. While studies have demonstrated reduction of SSIs in vascular patients from 33 to 13% with these adjuncts, infection rates remain high compared to other surgical fields [4]. The cost acquired with vascular surgical site infections is significant, with an added adjusted cost of approximately $11,000 and additional length of stay of 4.3 days per infection [5, 6].

Minimally invasive approaches across multiple surgical subspecialities have led to decreased incidence of surgical site infections, post-operative pain, and hospital length of stay. Single-port robot technology has demonstrated promising results in multiple surgical fields including decreased blood loss and shorter hospital stay in left liver lobectomy, decreased blood loss and shorter lengths of stay in colorectal surgery, and shorter operative times and less post-operative pain in cholecystectomy and inguinal hernia repair [7]. Given this, we aimed to investigate the feasibility of femoral artery dissection and anastomosis via a robotic single-port procedure from the mid-thigh in two cadavers with a beginner robotic vascular surgeon.

## Materials and methods

Two human cadaveric models were included in a 2-day workshop in a laboratory with fully implemented systems for research, development, and training. This study was carried out in accordance with ethical standards and the Declaration of Helsinki 1964; Institutional Review Board approval was not required as there were no living human subjects. One vascular surgeon was involved (AW), who completed a general surgery residency program with a formal 2-week robotics course and approximately 50 robotic surgery cases during residency. The surgeon had no robotic training during vascular surgery fellowship and at the time of the study had not participated in robotic surgery in 2 years. The surgeon was unfamiliar with the da Vinci SP surgical system, but was given a short training session on the platform prior to beginning the laboratory (Intuitive Surgical, Sunnyvale, CA, USA).

The procedure was performed in two human cadavers: an 80-year-old male with a BMI of 40 and a 75-year-old male with a BMI of 35.4. Both legs were used for dissection in the initial cadaver while only the left leg was utilized in the second because of the time allotted for the laboratory.

**Fig. 1:**
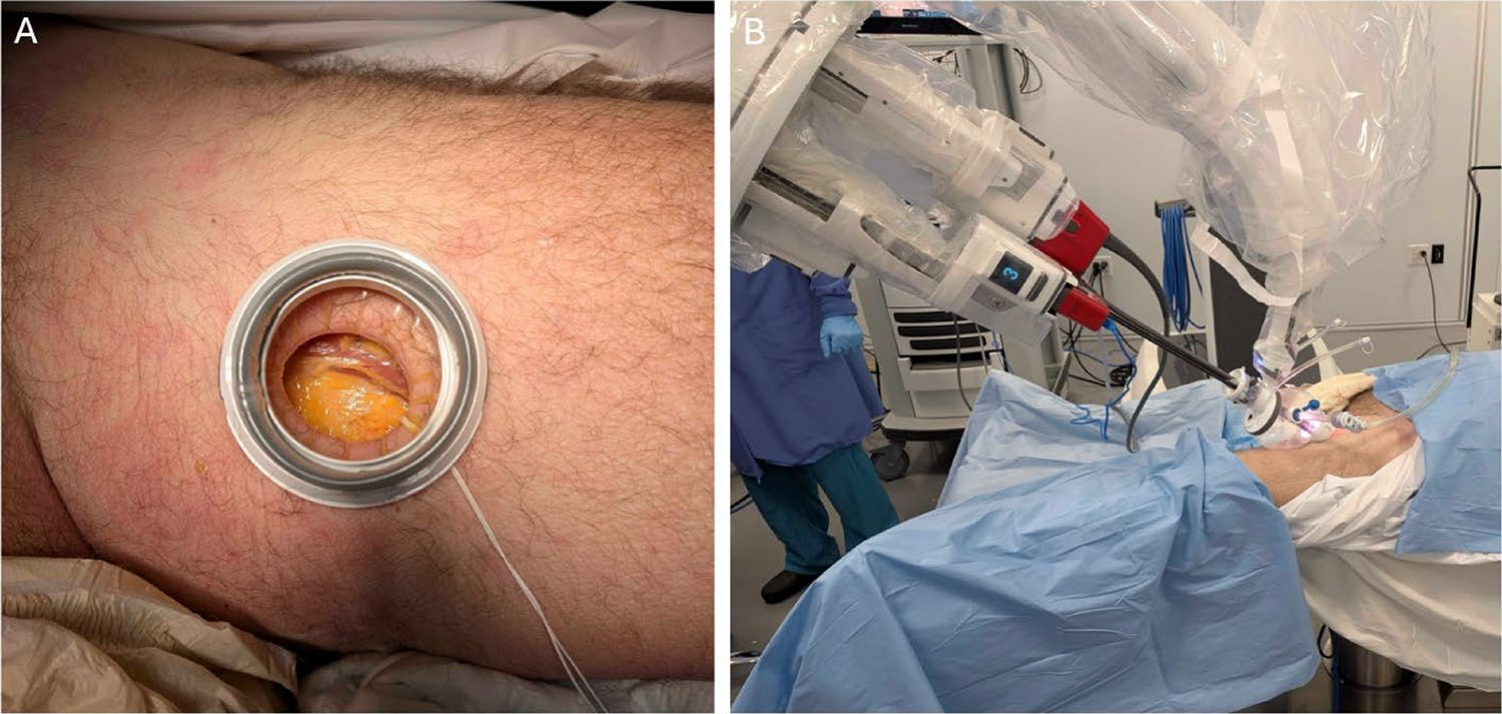
Incision, port placement, and docking. **A**: 3 cm mid-thigh incision with sartorius muscle exposed. **B**: SP system docked

All models were placed in the supine position on a split-leg table with the legs gently abducted. A 3 cm skin incision was made at the mid-thigh, 15 cm inferior to the inguinal ligament. The subcutaneous tissue was dissected with a combination of electrocautery and blunt dissection until the medial border of the sartorius muscle was identified and reflected laterally (Fig. 1A). The small SP Access Port (Intuitive Surgical, Sunnyvale, CA, USA) was then inserted and the SP Patient Cart docked. Notably, the inflated port allowed contact against the patient skin without pressure points (Fig. 1B). Carbon dioxide was insufflated to 12 mmHg to allow for initial subcutaneous dissection on the first cadaver and reduced to 10 mmHg on subsequent attempts, as no appreciable difference in dissection ability was noted at the lower pressure. The SP camera was initially configured for an upward view. Maryland bipolar forceps, monopolar curved scissors, and Cadiere forceps were used for the dissection.

**Fig. 2:**
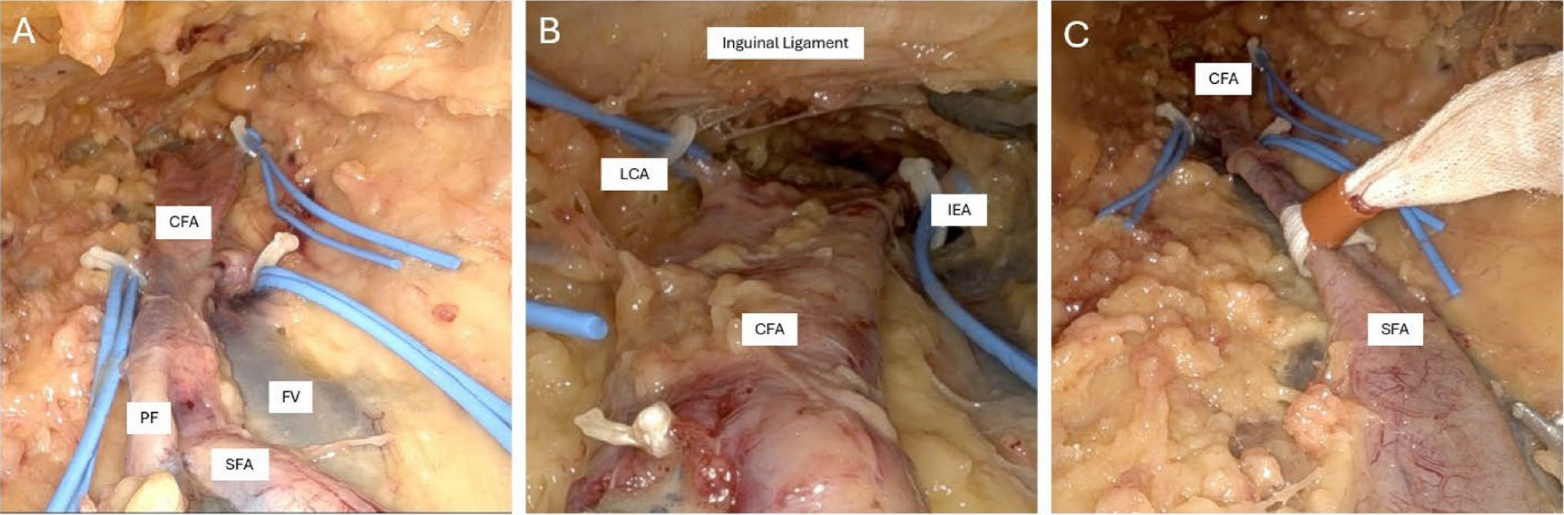
Intra-operative endoscope images. *CFA* Common femoral artery, *SFA* superficial femoral artery, *PF* profunda femoris artery, *FV* femoral vein, *IEA* inferior epigastric artery, *LCA* lateral circumflex artery. **A** Control of small vessels with silastic loops and Weck M-L Clips. **B** Control of SFA with umbilical tape and modified Rumel. **C** Dissection at the level of the inguinal ligament with isolation of the lateral circumflex and inferior epigastric arteries

The sartorius was used as the initial landmark with subcutaneous dissection along the medial border and reflection of the muscle laterally. The greater saphenous vein was visible in each leg and followed proximally toward the femoral vein and served as a medial landmark to dissection. The superficial femoral artery was identified and dissected initially anteriorly and proximally for dissection of the common femoral artery. Using sharp and monopolar dissection with the curved scissors, the superficial femoral artery, profunda femoris artery, and common femoral artery were dissected circumferentially. Smaller branches were controlled with silastic vessel loops cinched with plastic clips (Fig. 2A). Umbilical tape was cinched using a red rubber catheter similar to a Rumel tourniquet on the superficial femoral artery and profunda femoris (Fig. 2C). We propose proximal control in this model with endovascular balloon occlusion under fluoroscopy with sheath placement in the ipsilateral external iliac artery via the contralateral common femoral artery or brachial access, a common technique already used by vascular surgeons.

Dissection was carried proximally with clear view of the inguinal ligament superiorly. The lateral circumflex artery and inferior epigastric artery were dissected and controlled with vessel loops (Fig. 2B).

**Fig. 3:**
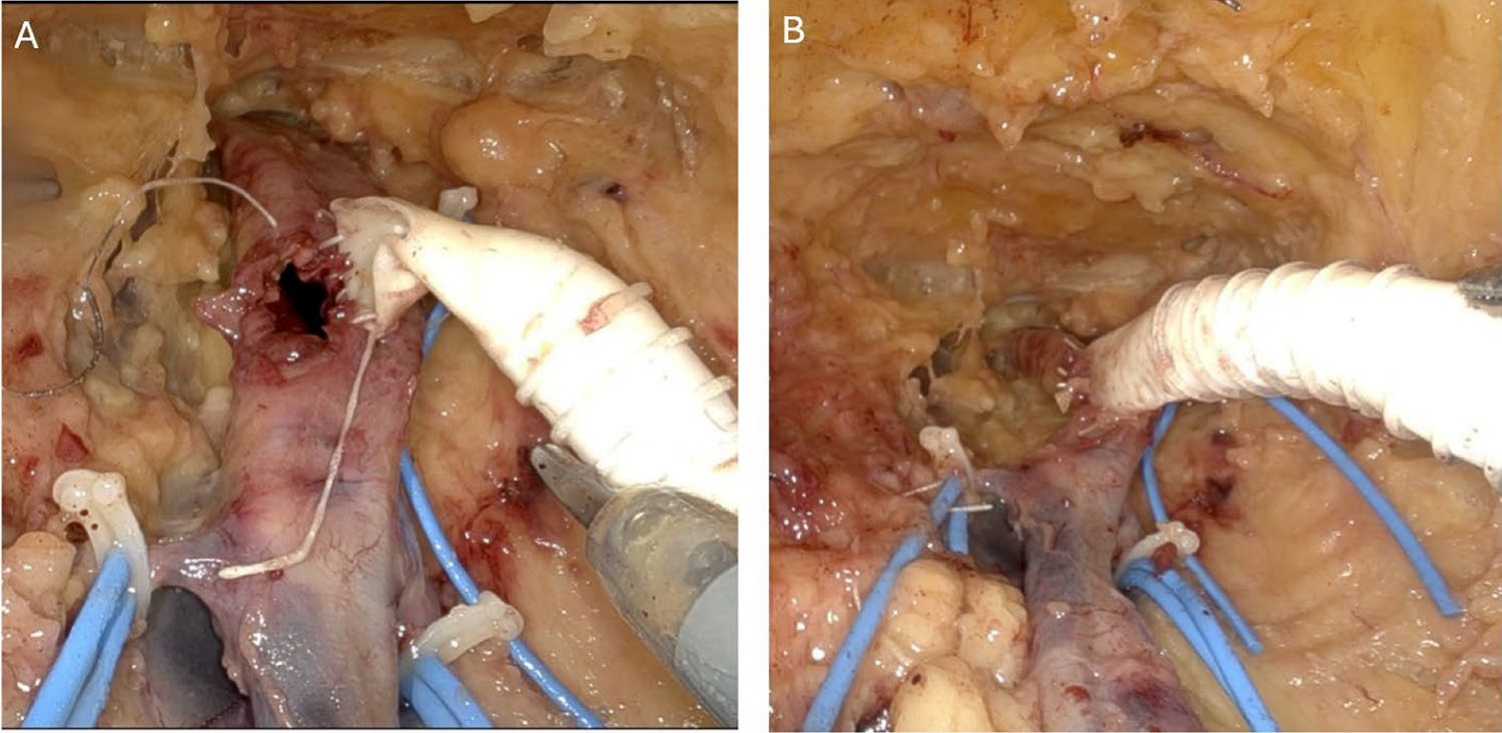
Creation of anastomosis. **A** Arteriotomy and partial anastomosis with 6-mm ringed PTFE **B** Completed anastomosis

Once the vessels were controlled with vessel loops or umbilical tape, a longitudinal arteriotomy was created with curved scissors and extended proximally and distally on the common femoral artery (Fig. 3A). Vascular anastomosis was created with 6-mm ringed PTFE via the access port and sutured using doubled-ended 20cm CV-5 Gore-Tex (Gore & Associates, Flagstaff, USA) suture for a running end to side anastomosis (Fig. 3B).

## Results

In all models, dissection of the femoral vessels and vascular anastomosis with 6-mm ringed PTFE was feasible. Importantly, proximal dissection under the inguinal ligament with visualization of the external iliac artery was accomplished, highlighting the extent of dissection that is possible from this approach. Table 1 summarizes the operative times for both dissection and anastomosis.

**Table 1:** Operative times

Attempt	Laterality	Cut down and port placement	Dissection for creation of vascular defect/otomy	Creation of proximal anastomosis	Total console time	Total insufflation time	Additional notes
1 (day 1)	Left	15 min	152 min	41min	230 min	238 min	
2 (day 1)	Right	5 min	55 min *	45 min	133 min	136 min	*Spent an additional 12 min post-anastomosis to get dissection up to the level of the epigastric
3 (day 2)	Left	10 min	56 min	42 min	110 min	111 min	
Mean		10 min	87 min	42.6 min	157.7 min	161.7 min	

**Cut down and port placement**—time from first incision to SP Access Port Kit fully docked

**Total console time**—time of first robotic movement by console surgeon to time of last motion (Note: breaks and interruptions are included as part of this time)

**Total insufflation time**—time from the beginning of CO_2_ insufflation to the end

**Mean**—mean times over the three attempts

Cut down and port placement was defined as time from incision to SP Access kit docking. Times ranged from 5 to 15 min, (mean 10 min, SD +/− 5 min). Time from initial dissection to creation of a common femoral arteriotomy ranged from 55 to 152 min (mean 87 min, SD +/− 55.7 min). Time for creation of the anastomosis ranged from 41 to 45 min (mean 42.6 min, SD +/− 2.08 min). Total console time ranged from 110 to 230 min (mean 157.7 min, SD +/− 63.7 min). Total insufflation time was similar and ranged from 111 to 238 min (mean 161.7 min, SD +/− 67.3 min).

Notably, the learning curve for dissection was quick with an initial dissection time of 152 min, which decreased to 55 min on second attempt. Vascular anastomosis took roughly 45 min on each attempt without a noticeable change in times between attempts. This may reflect a steeper learning curve for vascular anastomosis than dissection in this model.

## Discussion

Our study demonstrates the feasibility of femoral artery dissection and vascular anastomosis using a single-port incision at the mid-thigh. To date, vascular surgery has approached minimally invasive surgery through an endovascular approach. While endovascular revascularization is associated with decreased short-term mortality and wound infections, open procedures continue to show superior long-term patency and limb-associated adverse events [8, 9]. Robotic vascular surgery offers a minimally invasive approach to surgical bypass and the potential to reduce morbidity while maintaining the superior patency of open procedures.

Novel robotic approaches to vascular surgery have been recently described and appear to be a feasible bridge between endovascular and open vascular surgery. Watson et al. have recently described the use of the single-port system for upper and lower extremity vascular exposure and stent placement via robotic-assisted percutaneous access [10]. Similarly, robotic-assisted sutureless vascular anastomoses with PTFE grafts connected to self-expanding covered stents have also been described using a single-port technology [11]. Further, kidney transplantation and autotransplantation have been completed using single-port robotic-assisted platforms in humans with good short-term clinic results [12]. To the best of our knowledge, our described work is the first single-port sutured vascular anastomosis in the groin.

The technique appears to have an acceptable learning curve with a large decrease in operative time between the first and second attempt with a beginner robotic surgeon. The total operative insufflation time had an average of 161.7 min over the three attempts. This is similar in comparison to average operative times for open common femoral endarterectomies which are on average 146 ± 69.5 min based on NSQIP data [13]. Vascular anastomoses have been performed in humans using the robotic single-port system for kidney transplantation with vascular anastomoses ranging in time from 52 to 92 min in six patients, again similar to our average of 42.6 min for vascular anastomoses [12].

Surgical site infections remain one of the most common and costly complications of lower extremity bypass surgery, and a minimally invasive approach has consistently demonstrated decreased surgical site infections throughout surgical specialties [14]. The potential use of a single-port incision from the mid-thigh would eliminate the need for an infection-prone groin incision.

The disadvantage in the robotic approach is direct vascular control with surgical clamps. However, proximal control using an occlusion balloon is already a widely accepted practice in vascular surgery. Small vessels can be controlled with silastic vessel loops and synched plastic clips. In our model, both the superficial femoral and profunda femoris arteries were controlled with umbilical tape and a modified Rumel technique with a cut red rubber catheter and plastic clip. It is possible that small 5 mm incisions would be needed to place surgical clamps directly on these larger vessels for control, a technique already described in the robotic vascular surgery experience from Europe [15].

The largest limitation of our trial is that the work was completed in cadaver models without a reliable method to assess vascular control and hemostasis. Several animal models including porcine have too small a femoral canal with limited subcutaneous tissue to adequately model human dissection and anatomy. Additional feasibility work is required to better understand this technique. Live porcine models with iliac artery anastomoses to assess for hemostatic anastomoses with the SP system are planned. Vascular surgery remains without a Food and Drug Administration indication for da Vinci SP in the USA and will require an FDA-approved Investigational Device Exemption to study clinically. As a result, a Physician-Sponsored Investigational Device Exemption for the SP robot and femoral artery dissection is in progress in addition to an IRB at the University of Chicago with plans for a small first-in-human trial at our institution.

## Fundings

This work was supported by Intuitive Surgical Inc., Sunnyvale, CA, USA, which provided financial support with cadavers and technology resources for the experimental studies.

## Data Availability

No datasets were generated or analyzed during the current study.
